# Systemic Inflammation and Malnutrition Define a High-Risk Phenotype in Chronic Limb-Threatening Ischemia

**DOI:** 10.3390/jcm15103987

**Published:** 2026-05-21

**Authors:** Paula Luque-Linero, Prado Salamanca-Bautista, Eduardo Carmona-Nimo, Teresa Arrobas-Velilla, Francisco José Rivera-de-los-Santos, Miguel Ángel Rico-Corral

**Affiliations:** 1 Department of Internal Medicine, Hospital Universitario Virgen Macarena, 41009 Seville, Spain; pradosalamanca@gmail.com (P.S.-B.); eduardom.carmona.sspa@juntadeandalucia.es (E.C.-N.); marc@us.es (M.Á.R.-C.); 2 Department of Medicine, University of Seville, 41009 Seville, Spain; 3 Department of Biochemistry, Hospital Universitario Virgen Macarena, 41009 Seville, Spain; teresa.arrobas.sspa@juntadeandalucia.es; 4 Department of Experimental Psychology, University of Seville, 41004 Seville, Spain; franciscorivera@us.es

**Keywords:** critical limb ischemia, IL-6, hsCRP, malnutrition, inflammatory biomarkers

## Abstract

**Objective:** This study aimed to evaluate the prognostic value of inflammatory biomarkers and their interaction with nutritional status for risk stratification in patients with chronic limb-threatening ischemia (CTLI). **Material and Methods:** This was a prospective, single-center observational cohort study including adult patients admitted with CTLI. Clinical outcomes included major amputation, major vascular events (MACE), and all-cause mortality. Multivariate logistic regression analyses were performed using two separate models, one including IL-6 and another including hsCRP, to avoid potential collinearity between biomarkers. Model discrimination was assessed using ROC curves, and Kaplan–Meier survival analyses were performed. **Results:** A total of 170 patients were included (mean age 72 ± 12 years; 74% male), with high cardiovascular risk and frequent malnutrition and sarcopenia. At 6 months, major amputations occurred in 35.3% of patients, MACE in 35%, and all-cause mortality in 32%. In multivariable analyses, malnutrition was the strongest independent predictor of the composite endpoint. IL-6 (OR 2.90, 95% CI 1.45–5.81; *p* = 0.003) and hsCRP values above the median (OR 4.22, 95% CI 2.04–8.72; *p* < 0.001) remained independently associated with adverse outcomes, together with age > 72 years. The hsCRP-based model showed slightly higher discriminative performance than the IL-6 model (AUC = 0.77 VS AUC = 0.75). Kaplan–Meier analyses demonstrated significantly reduced event-free survival in patients with elevated inflammatory biomarkers. **Conclusions**: In CTLI, systemic inflammation and nutritional status jointly identify patients at extremely high risk of adverse outcomes. hsCRP, given its availability, may be a practical tool for clinical risk stratification.

## 1. Introduction

Chronic limb-threatening ischemia (CLTI) represents the most advanced stage of peripheral arterial disease and is associated with a markedly poor prognosis, including high rates of major amputation and mortality despite contemporary medical and revascularization strategies. Patients with CLTI frequently present with a heavy burden of comorbidities, advanced age, and functional impairment, making early risk stratification a critical but still unresolved clinical challenge [1].

Beyond traditional cardiovascular risk factors, increasing evidence supports the role of systemic inflammation as a key driver of atherosclerosis progression and adverse outcomes [2]. Although several biomarkers have been proposed to reflect these mechanisms [3,4], inflammatory biomarkers such as hsCRP and IL-6 remain among the most clinically accessible and consistently associated with adverse cardiovascular and functional outcomes [5]. High-sensitivity C-reactive protein (hsCRP) has been widely validated as a prognostic biomarker in cardiovascular disease [6,7,8], while interleukin-6 (IL-6), an upstream mediator of the inflammatory cascade, has emerged as a potential therapeutic target [9]. However, data on the prognostic value of these biomarkers in CLTI, particularly in the acute hospital setting, remain limited.

In parallel, malnutrition and sarcopenia are highly prevalent in patients with advanced vascular disease and have been consistently associated with worse clinical outcomes, including impaired wound healing, increased risk of amputation, and mortality [10,11,12,13,14]. Sarcopenia in patients with CTLI is considered a multifactorial process involving chronic inflammation, oxidative stress, skeletal muscle mitochondrial dysfunction, and dysregulation of anabolic and catabolic signaling pathways [15]. Importantly, malnutrition in this context often reflects a chronic inflammatory and catabolic state rather than isolated nutritional deficiency, suggesting a close interaction between systemic inflammation, muscle degradation, and metabolic deterioration [16].

Despite these observations, the combined impact of inflammatory biomarkers and nutritional status on prognosis in patients with CLTI has not been fully characterized. Understanding this interplay may help to identify a subgroup of patients at particularly high risk, in whom more intensive monitoring and tailored therapeutic strategies could be considered.

Therefore, the aim of this study was to evaluate the prognostic value of IL-6 and hsCRP and to explore their relationship with nutritional status for risk stratification in patients hospitalized with CTLI.

## 2. Methods

### 2.1. Study Design

This was a prospective, single-center hospital-based observational cohort study with descriptive and analytical scope. The prognostic value of emerging biomarkers in CTLI was assessed in relation to amputation rates, occurrence of vascular events (stroke or myocardial infarction), and all-cause mortality. The study was designed in accordance with the STROBE guidelines to ensure the quality of observational research [17].

### 2.2. Study Population

#### 2.2.1. Inclusion Criteria

Adult men and women aged 18 years or older admitted to the Internal Medicine ward with CTLI were included, regardless of whether the diagnosis was de novo or previously established. CTLI was defined by rest pain lasting at least two weeks and/or the presence of trophic lesions, irrespective of the ankle–brachial index value [18,19]. All patients had a Doppler ultrasound performed prior to or within the first 24 h of admission consistent with this diagnosis. Only cases of atherosclerotic etiology were included.

#### 2.2.2. Exclusion Criteria

Patients with CTLI due to non-atherosclerotic causes, those who did not provide informed consent, and those in whom blood samples were obtained more than 72 h after admission or after any intervention (amputation or revascularization) were excluded. In addition, patients with infected trophic lesions or evidence of osteomyelitis were also excluded.

### 2.3. Variables

Demographic variables collected from clinical records included sex, ethnicity (predominantly Caucasian), postal code, and place of residence (urban or rural). Clinical data encompassed major cardiovascular risk profiles and chronic conditions, such as tobacco exposure, elevated blood pressure, lipid metabolism disorders, type 2 diabetes mellitus, excess body weight assessed by body mass index, and a family history of premature cardiovascular disease. Chronic kidney disease was defined by an estimated glomerular filtration rate below 60 mL/min/1.73 m^2^ or an albumin-to-creatinine ratio greater than 30 mg/g, persisting for more than three months. Anemia was defined according to hemoglobin levels < 13 g/dL [20]. Functional status was evaluated using the Barthel Index, with severe dependency defined as a score < 40. Nutritional status was assessed using the Mini Nutritional Assessment–Short Form (MNA-SF), classifying patients as well nourished, at risk of malnutrition, or malnourished, while sarcopenia risk was evaluated using the SARC-F questionnaire, with scores ≥ 4 indicating high risk. Major adverse cardiovascular events (MACE) were defined as the composite of myocardial infarction, cerebrovascular ischemia, and mortality. Major adverse limb events (MALE) included major amputation, need for repeat revascularization, or restenosis-related complications. Major amputation was defined as any amputation above the ankle level, whereas minor amputation included distal amputations below the ankle. In addition, a combined outcome variable was analyzed, including MACE and major amputation.

### 2.4. Laboratory Determinations

Blood and urine samples were collected within the first 72 h of admission, prior to any intervention, by fasting venipuncture at 7:30 a.m. Routine analyses were processed using standardized methods in the hospital biochemistry laboratory.

An additional biochemistry tube was used to obtain serum for biomarker analysis. Samples were centrifuged within two hours of collection at 3500 rpm for 10 min, aliquoted (three 0.5 mL aliquots), labeled according to biobank protocols, and stored at −80 °C until analysis, with support from the hospital biobank platform.

IL-6 determination was performed in vitro using the ELECSYS IL-6 electrochemiluminescence immunoassay on a previously calibrated cobas e immunoanalyzer. Patient serum samples stored at −80 °C were used, and instrument calibration was performed prior to analysis.

The assay, with a total duration of 18 min, is based on a sandwich immunoassay principle. The sample was incubated with a biotinylated anti-IL-6 monoclonal antibody, followed by the addition of a ruthenium-labeled anti-IL-6 monoclonal antibody and streptavidin-coated microparticles, forming an immune complex. The reaction mixture was then transferred to the measuring cell, where the complexes were magnetically captured on the electrode surface. After washing to remove unbound components, a controlled electrical current induced a chemiluminescent reaction, and the emitted light was measured by a photomultiplier.

IL-6 concentrations were automatically calculated by the analyzer using an instrument-specific calibration curve derived from a two-point calibration and a master curve provided via cobas link, and results were expressed in pg/mL.

High-sensitivity C-reactive protein (hs-CRP) was determined using immunological techniques (ELISA). This method employs specific antibodies that bind to CRP, generating a colorimetric or chemiluminescent reaction to enable its quantification. It is a latex immunoassay developed to provide accurate and reproducible measurements of blood CRP concentrations in serum and plasma. Agglutination occurs as a result of the antigen–antibody reaction between CRP present in the sample and anti-CRP antibodies adsorbed onto latex particles. This agglutination is detected as a change in absorbance at 572 nm, with the rate of change being proportional to the amount of CRP in the sample. The analysis was performed using an Alinity c analyzer.

### 2.5. Statistical Analysis

Descriptive statistics were used to summarize baseline clinical and laboratory characteristics. A descriptive subgroup analysis was performed according to the occurrence or absence of the event. Continuous variables were expressed as mean ± standard deviation or median and interquartile range, depending on data distribution, while categorical variables were reported as frequencies and percentages. For analytical purposes, and in the absence of established reference cut-off values for this clinical context, IL-6 and hs-CRP concentrations were dichotomized based on their median values. Additionally, an exploratory survival-based analysis using the survival cutpoint function from the survminer package in R was performed to identify the optimal prognostic threshold for IL-6 and hs-CRP according to the log-rank statistics. The optimal cut-off identified was very close to the median value observed in the cohort; therefore, the median was ultimately selected for the main analyses to provide a more balanced distribution between groups and improve statistical robustness.

Group comparisons were performed using Student’s *t* test or the Mann–Whitney *U* test for continuous variables, and the chi-square or Fisher’s exact test for categorical variables.

Multivariate analyses were conducted using logistic regression models to identify independent predictors of amputation, vascular events, and all-cause mortality. To account for potential collinearity between interleukin-6 (IL-6) and high-sensitivity C-reactive protein (hsCRP), given the role of IL-6 as an upstream regulator of CRP synthesis [21], two separate multivariable models were constructed: one including IL-6 and another including hsCRP. A *p* value < 0.05 was considered statistically significant.

The prognostic performance was evaluated using receiver operating characteristic (ROC) curve analysis performed separately for each predictive model. The area under the curve (AUC) was calculated to assess discriminative ability, and optimal cut-off values were identified.

Survival and event-free analyses were conducted using Kaplan–Meier curves, with comparisons assessed by the log-rank test.

### 2.6. Ethics Statement

This study was conducted in accordance with general and specific ethical principles concerning the right to privacy, anonymity, confidentiality, data protection, and the right to information. The protocol was approved by the Clinical Research Ethics Committee under Spanish Biomedical Research Law 14/2007.

The study was conducted in accordance with the Declaration of Helsinki and approved by the Ethics Committee of Hospitales Universitarios Virgen Macarena y Virgen del Rocío (protocol code 1293-N-23; approval date: 28 November 2023) for studies involving human participants. Written informed consent was obtained from all participants prior to inclusion.

Investigators adhered strictly to the study protocol and data collection procedures. The study was carried out in compliance with the Declaration of Helsinki and Organic Law 3/2018 on Personal Data Protection and digital rights. All clinical data were anonymized.

## 3. Results

### 3.1. Baseline Characteristics and Laboratory Findings

A total of 170 patients were included in the analysis, with a mean age of 72 ± 12 years; 58.2% lived in rural areas. Cardiovascular risk factors were highly prevalent, including hypertension (83.5%), type 2 diabetes mellitus (75.9%), dyslipidemia (59.4%), smoking history (65.6%), and obesity (14.1%). Comorbidities were frequent, particularly anemia (75.3%), malnutrition (50.6%), sarcopenia (45.3%), chronic kidney disease (30.6%), atrial fibrillation (25.9%), ischemic heart disease (28.2%), and previous stroke (24.7%).

Patients who experienced a composite outcome, defined as major amputation, major vascular events (stroke or myocardial infarction), or death, were older and exhibited a higher prevalence of depression (18.8% vs. 7.8%), malnutrition (68.8% vs. 34.4%), sarcopenia (57.5% vs. 34.4%), and history of malignancy (27.3% vs. 14.3%), as well as poorer functional status, reflected by lower Barthel Index scores (median 60 vs. 80), compared with those without the composite outcome.

The remaining comorbidities and biomarkers are summarized in Table 1.

### 3.2. Events at 6-Month Follow-Up

At 6-month follow-up, adverse outcomes were frequent. Amputation occurred in 58.4% of patients, including 23.1% minor and 35.3% major amputations, representing a 10% increase compared with 3-month rates. Endovascular revascularization was performed in 44.5%, while 35.3% were managed conservatively. Major adverse limb events (MALE) occurred in 74% of patients, and major adverse cardiovascular events (MACE) in 35.3%. Ischemic heart disease events were observed in 8.7%, stroke in 2.3%, and stent restenosis in 5.8%. Readmission due to CTLI occurred in 34.7%. All-cause mortality at 6 months reached 31.6%, and the combined endpoint of MACE plus amputation was observed in 61.8% of patients.

### 3.3. Univariate and Multivariate Analysis

In univariate analyses, several clinical and biochemical variables were associated with the composite endpoint of major amputation, major adverse cardiovascular events (MACE), or all-cause mortality. Older age (>72 years), male sex, depression, sarcopenia, anemia, and malnutrition were associated with poorer outcomes. Among laboratory parameters, elevated inflammatory markers (IL-6 and hsCRP), hypoalbuminemia, and the CRP/albumin ratio showed significant associations with the composite endpoint (Table 2).

To avoid collinearity between IL-6 and hsCRP, two separate multivariable logistic regression models were constructed, adjusted for age > 72 years, sex, malnutrition, sarcopenia, anemia, and LDL cholesterol (Table 3 and Table 4).

**Figure 1:**
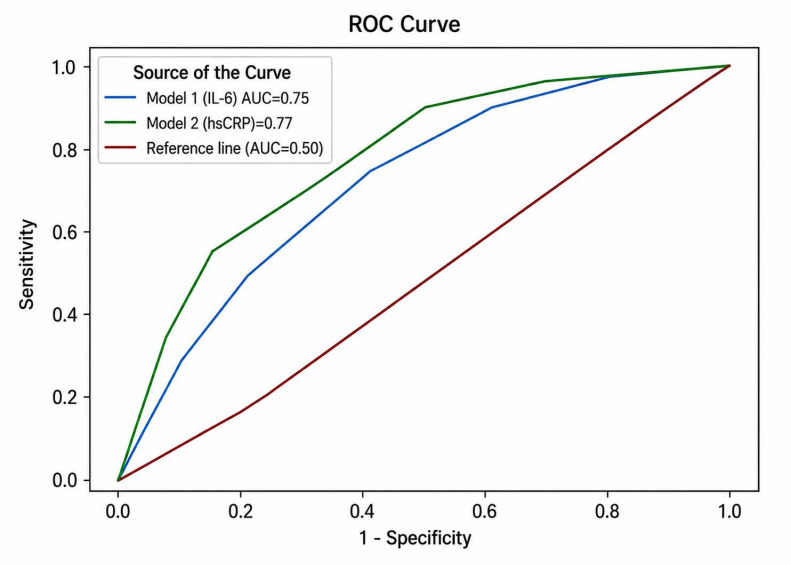
Receiver operating characteristic (ROC) curves comparing the predictive performance of the multivariate models for adverse outcomes (major amputation, major adverse cardiovascular events, or mortality). Model 1 included IL-6 as the inflammatory biomarker, while Model 2 included hsCRP. The reference line represents the performance of a non-discriminatory model (AUC = 0.5). ROC: Receiver operating characteristics curve; AUC: area under the curve.

In both models, malnutrition was the strongest independent predictor of adverse outcomes. In the IL-6 model, malnutrition (OR 4.25, *p* < 0.001), IL-6 above the median (OR 2.90, *p* = 0.003), and age > 72 years (OR 2.44, *p* = 0.012) remained independently associated with the composite endpoint (AUC 0.750). Similarly, in the hsCRP model, malnutrition (OR 4.68, *p* < 0.001), hsCRP above the median (OR 4.22, *p* < 0.001), and age > 72 years (OR 2.19, *p* = 0.031) were independent predictors, with slightly higher discriminative performance (AUC 0.775) (Figure 1).

**Figure 2:**
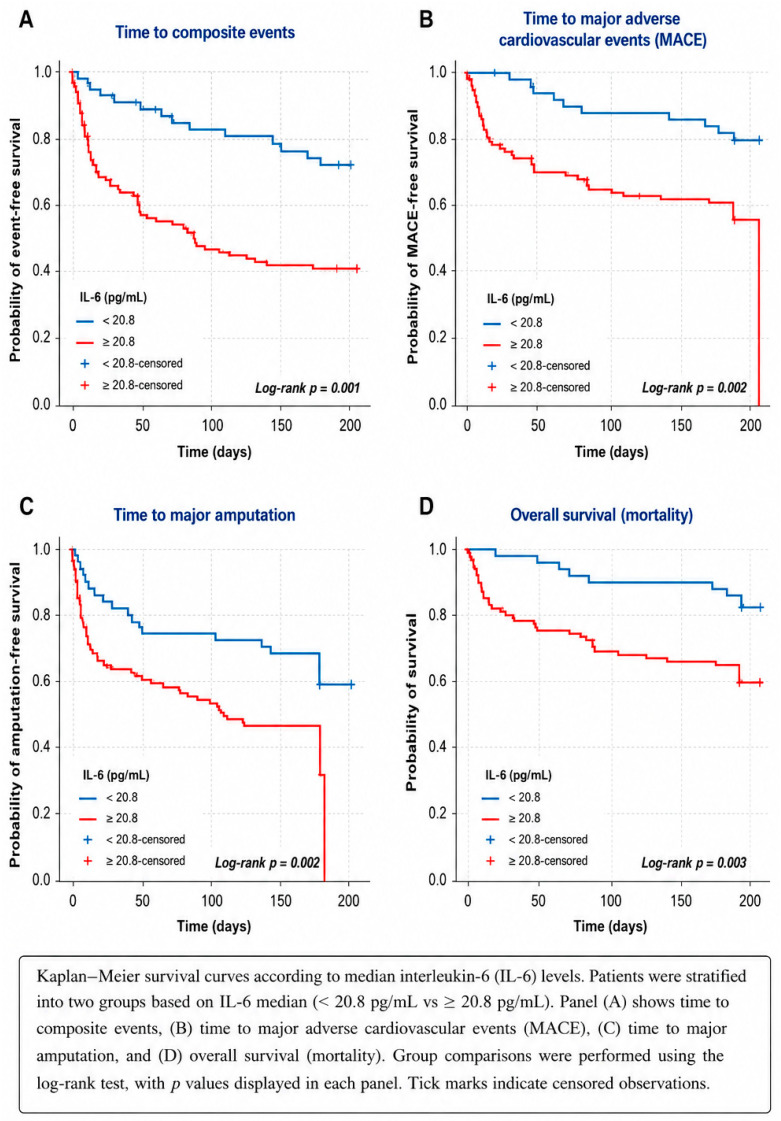
Kaplan–Meier curves according to median interleukin-6 (IL-6) levels.

**Figure 3:**
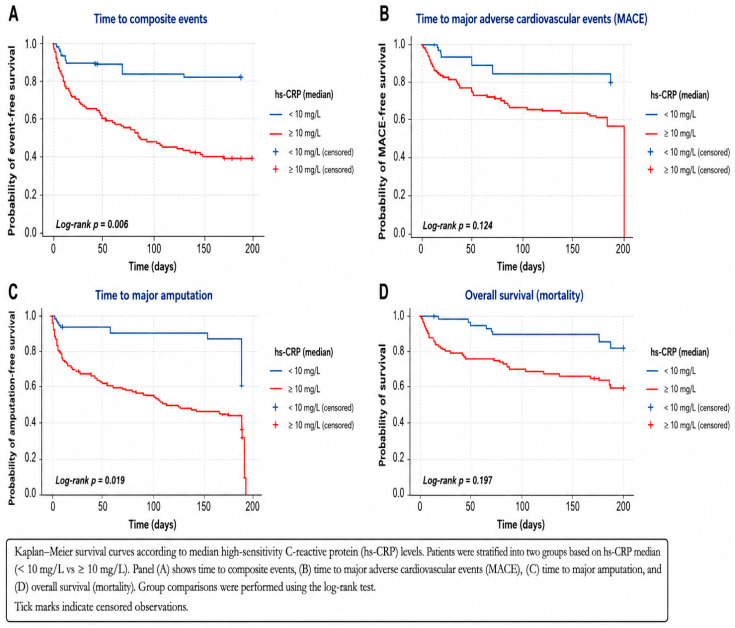
Kaplan–Meier survival curves according to median high-sensitivity C-reactive protein (hs-CRP) levels.

Kaplan–Meier survival analyses showed early and sustained separation of curves according to IL-6 and hsCRP levels. Elevated IL-6 and hsCRP were associated with significantly lower event-free survival for the composite endpoint and worse all-cause survival. While IL-6 showed only a non-significant trend toward increased MACE, elevated hsCRP was significantly associated with higher MACE incidence. Both biomarkers were significantly associated with reduced amputation-free survival (Figure 2 and Figure 3).

## 4. Discussion

In this prospective cohort of patients hospitalized with CTLI, we found that systemic inflammation and nutritional status jointly identify a population at extremely high risk of adverse outcomes. Both IL-6 and hsCRP were independently associated with 6-month prognosis, including major amputation, major adverse cardiovascular events, and all-cause mortality, reinforcing the role of inflammation as a central determinant of disease severity in this setting.

Our findings are consistent with previous studies demonstrating the prognostic value of inflammatory biomarkers in cardiovascular diseases [6,22]. However, patients in our cohort exhibited markedly higher levels of IL-6 and hsCRP compared with most prior reports [23,24,25,26,27]. This likely reflects the advanced stage of disease and the acute clinical condition at the time of hospital admission. Importantly, these elevated levels cannot be attributed to infection, as patients with active infectious processes were excluded. Rather, they suggest a profound inflammatory activation inherent to advanced CLTI. Despite this acute context, the consistent association between inflammatory biomarkers and adverse outcomes supports their pathophysiological relevance rather than a purely transient response.

Although IL-6 and hsCRP showed comparable prognostic performance, hsCRP demonstrated slightly better discriminative capacity and remains more readily applicable in routine clinical practice. Its widespread availability, lower variability, and strong analytical standardization support its use as a practical tool for risk stratification. In contrast, IL-6 measurement is currently limited by technical and logistical constraints and does not appear to provide substantial incremental value over hsCRP in this clinical setting [2].

Nevertheless, IL-6 may gain clinical relevance in the near future [28]. As an upstream mediator of the inflammatory cascade, it represents a promising therapeutic target, and ongoing clinical trials evaluating IL-6 pathway inhibitors may redefine its role from a prognostic biomarker to a treatment-guided marker [29]. This evolving landscape could increase the clinical utility of IL-6 assessment in selected high-risk patients.

Although IL-6 and hsCRP showed the strongest association with adverse outcomes in our cohort, other inflammatory markers may also provide relevant prognostic information in patients with CLTI. In our study, the neutrophil-to-lymphocyte ratio was not included in the final prognostic models because it did not demonstrate significant prognostic value in our cohort, possibly due to differences in disease severity, acute clinical presentation, or sample characteristics compared with previous reports [30]. In addition, biomarkers reflecting other pathophysiological pathways, such as markers related to oxidative stress and muscle metabolism, could potentially contribute to improving future multimarker risk stratification models in this high-risk population [31,32].

A key finding of our study is the strong prognostic impact of malnutrition, which emerged as the most powerful independent predictor of adverse outcomes. While our previous work focused on sarcopenia as an independent prognostic factor in CLTI using anthropometric and functional assessment tools [15], the present study expands these observations by exploring the complementary role of systemic inflammation and its interaction with nutritional and metabolic deterioration. This observation is in line with previous reports linking poor nutritional status to increased mortality, impaired wound healing, and higher amputation rates in patients with advanced vascular disease [33,34,35]. Importantly, our results highlight that malnutrition in CLTI should not be interpreted solely as inadequate nutritional intake but rather as a manifestation of a chronic inflammatory and catabolic state.

The interaction between inflammation and malnutrition appears to be central to disease progression. Chronic inflammatory activation, driven by cytokines such as IL-6, promotes a catabolic environment characterized by increased energy expenditure, anabolic resistance, and muscle protein breakdown. This leads to progressive sarcopenia and functional decline, further increasing vulnerability to adverse clinical events. In parallel, inflammation suppresses the synthesis of visceral proteins such as albumin, reinforcing the concept that commonly used nutritional markers may primarily reflect disease severity rather than true nutritional status.

This bidirectional relationship establishes a self-perpetuating cycle in which inflammation and malnutrition amplify each other, ultimately contributing to poor outcomes. In this context, inflammatory biomarkers such as IL-6 and hsCRP may serve not only as indicators of risk but also as markers of an active pathophysiological process linking vascular disease, metabolic deterioration, and functional impairment [36].

Finally, age and age-related chronic low-grade inflammation, commonly referred to as “inflammaging,” may further amplify this vicious cycle [16]. Aging is associated with a persistent pro-inflammatory state characterized by increased circulating levels of cytokines such as IL-6 and C-reactive protein, which play a central role in the development and progression of atherosclerosis [37,38]. In the context of CTLI, inflammaging may exacerbate arterial narrowing and impair tissue perfusion, contributing to disease progression and worse clinical outcomes. Our findings support this concept, highlighting the interplay between aging, inflammation, and vascular pathology.

This study has several limitations. It is a single-center study conducted in patients with advanced disease during the acute phase, which may limit the generalizability of the results. In addition, the relatively limited sample size may have reduced the statistical power to detect smaller effects and precluded more extensive subgroup analyses. Furthermore, the cut-off values used for biomarker categorization were derived from the study cohort and may therefore have limited external applicability; consequently, these thresholds should be validated in independent populations. Finally, although variables related to disease severity, including Fontaine classification and anatomical distribution of arterial disease, were considered in the analyses, detailed treatment-related variables, particularly pharmacological therapies and specific revascularization strategies, were not systematically available for all patients and therefore could not be fully incorporated into the multivariate models. These factors may influence both inflammatory biomarkers and clinical outcomes and should be addressed in future prospective studies with more comprehensive therapeutic characterization.

However, the study also has notable strengths. Evidence addressing inflammation in chronic limb-threatening ischemia remains limited, and our work provides valuable real-world insight by evaluating two pro-inflammatory biomarkers in this clinical context. In addition, a major strength and novel aspect of this study is the integration of systemic inflammation and malnutrition as interconnected pathophysiological processes, providing a more comprehensive approach to risk assessment in this high-risk population.

## 5. Conclusions

Systemic inflammation and malnutrition are key determinants of prognosis in patients with CTLI. Malnutrition, likely reflecting an underlying inflammatory and catabolic state, was strongly associated with adverse outcomes. Both hsCRP and IL-6 independently predicted risk, with hsCRP representing a practical tool for clinical stratification.

## Figures and Tables

**Table 1:** Clinical characteristics and biomarkers of the study population according to the composite event.

Baseline Characteristics	Total Population	Composite Outcome Yes	Composite Outcome No	*p*
	(n = 170)	(n = 107)	(n = 63)	
Age (years)	72 ± 12	73 ± 11	68 ± 11	0.15
Place of residence				
Rural area, n (%)	99 (58.2)	45 (56.3)	54 (60.0)	0.62
Vascular risk factors, n (%)				
Hypertension	142 (83.5)	69 (86.3)	73 (81.1)	0.37
Type 2 diabetes mellitus	129 (75.9)	58 (72.5)	71 (79.0)	0.33
Dyslipidemia	101 (59.4)	42 (52.5)	59 (65.6)	0.08
Smoking status, n (%)				
Current or former smoker	113 (65.6)	49 (61.3)	64 (71.1)	0.17
Obesity (BMI > 30 kg/m^2^)	24 (14.1)	10 (22.5)	14 (22.2)	0.96
Other medical history, n (%)				
Alcohol use disorder	46 (27.1)	22 (27.5)	24 (26.7)	0.9
Ischemic heart disease	48 (28.2)	19 (23.8)	29 (32.2)	0.22
Previous stroke	42 (24.7)	20 (25.0)	22 (24.4)	0.93
Chronic kidney disease	52 (30.6)	26 (32.5)	26 (28.9)	0.61
Preserved LVEF	36 (21.2)	19 (23.8)	17 (18.9)	0.61
Atrial fibrillation	44 (25.9)	24 (30.0)	20 (22.2)	0.25
Autoimmune disease	10 (5.9)	4 (5.0)	6 (6.7)	0.64
COPD	19 (11.2)	10 (13.8)	9 (11.2)	0.77
Depression	22 (12.9)	15 (18.8)	7 (7.8)	0.03
Malnutrition	86 (50.6)	55 (68.8)	31 (34.4)	<0.001
Sarcopenia	77 (45.3)	46 (57.5)	31 (34.4)	0.03
History of malignancy	30 (17.6)	12 (27.3)	18 (14.3)	0.05
Obstructive sleep apnea	13 (7.6)	5 (6.3)	8 (8.9)	0.52
Anemia	128 (75.3)	60 (75.0)	68 (75.6)	0.93
Scales				
Barthel Index, median (IQR)	80 (40–96.2)	60 (20–80)	80 (50–75)	<0.001
SARC-F, mean ± SD	5.36 ± 2.86	6 ± 2	4 ± 2	<0.001
MNA-SF, mean ± SD	7.88 ± 3.32	7 ± 3	8 ± 3	<0.001
Biomarkers (Median, IQR)				
hsCRP	81.8 (28.62–132)	99.8 (78.5–128.5)	52 (7–97)	0.03
IL-6	38.4 (19.25–66.6)	42.7 (12.7–772.7)	26.3 (7.3–45.3)	0.03
Albumin (g/dL)	3 (2.87–3.8)	3.1 (2.75–3.55)	3.6 (3.1–3.9)	0.62
Prealbumin (mg/dL)	8.97 (13.2–17.8)	12 (7.92–15.7)	14.4 (11.1–19.7)	0.45
LDL cholesterol	72 (56–89)	68 (55.5–84)	77 (56.5 -101)	0.35
HDL cholesterol	30 (24–37)	28 (23–36)	31 (25.5–37)	0.01
Neutrophil-to-lymphocyte ratio	4.15 (2.53–6.7)	4.73 (3.08–8.2)	3.36 (2.11–5.6)	0.02
Lymphocyte-to-monocyte ratio (LMR)	1.9 (1.31–2.78)	1.79 (1.28–2.44)	1.79 (1.4–2.26)	0.47
Platelet-to-lymphocyte ratio (PLR)	180 (123–271)	180 (137.7–309)	179.4 (114–226)	0.1
Monocyte-to-HDL ratio	29.29 (19.9–37.6)	31 (21.2–40.5)	27 (17.9–36.7)	0.04
Triglyceride–glucose index	1.05 (0.73–1.44)	1.11 (0.71–1.51)	1 (0.76–1.4)	0.97
hsCRP/albumin ratio	24.2 (7.18–43.5)	33.2 (11.8–65.1)	16 (3.3–31.35)	0.01

COPD, chronic obstructive pulmonary disease; hsCRP, high-sensitivity C-reactive protein; IL-6, interleukin-6; LVEF, left ventricular ejection fraction; LDL, low-density lipoprotein; HDL, high-density lipoprotein; LMR, lymphocyte-to-monocyte ratio; MNA-SF, Mini Nutritional Assessment–Short Form; PLR, platelet-to-lymphocyte ratio. SARC-F, Strength, Assistance with walking, Rise from a chair, climb stairs, and Falls questionnaire. IQR: Interquartile Range. SD: Standard Deviation.

**Table 2:** Univariate analysis using logistic regression for mortality, MACE, and major amputation.

Variable	OR (95% CI)	*p* Value
Demographic characteristics		
Age > 72 years	2.46 (1.31–4.64)	0.005
Male sex	2.12 (1.01–4.44)	0.04
Rural residence	1.02 (0.54–1.91)	0.95
Cardiovascular risk factors		
Hypertension	2.01 (0.85–4.69)	0.11
Type 2 diabetes mellitus	0.87 (0.42–1.79)	0.7
Dyslipidemia	0.65 (0.34–1.22)	0.17
Obesity	0.94 (0.45–1.97)	0.86
Current smoking	0.61 (0.31–1.19)	0.14
Comorbidities		
Ischemic heart disease	0.65 (0.33–1.29)	0.21
Stroke	1.22 (0.60–2.50)	0.58
Chronic kidney disease	1.11 (0.57–2.15)	0.76
Heart failure	1.23 (0.64–2.38)	0.53
Atrial fibrillation	1.57 (0.77–3.21)	0.23
COPD	0.95 (0.38–2.32)	0.9
Depression	2.27 (0.87–5.90)	0.09
Sarcopenia	2.51 (1.33–4.75)	0.01
Anemia	3.12 (1.47–6.60)	0.03
History of malignancy	1.31 (0.59–2.91)	0.51
Inflammatory and nutritional parameters		
hsCRP ≥ 81.8 mg/L	5.02 (1.60–15.76)	0.01
IL-6 ≥ 20.8 pg/mL	3.33 (1.65–6.73)	<0.001
Malnutrition	4.00 (2.08–7.70)	<0.001
Albumin (g/dL)	0.25 (0.13–0.48)	<0.001
Prealbumin (mg/dL)	0.58 (0.31–1.09)	0.093
Lipid profile		
LDL cholesterol	0.47 (0.25–0.89)	0.02
HDL cholesterol	0.64 (0.35–1.20)	0.16
Inflammatory indices		
Neutrophil-to-lymphocyte ratio	1.64 (0.88–3.06)	0.11
Lymphocyte-to-monocyte ratio (LMR)	0.58 (0.31–1.08)	0.08
Platelet-to-lymphocyte ratio (PLR)	1.11 (0.60–2.05)	0.74
Monocyte-to-HDL ratio	1.73 (0.93–3.22)	0.08
Triglyceride–glucose index	0.93 (0.48–1.78)	0.82
hsCRP/albumin ratio	3.22 (1.69–6.12)	<0.001

COPD, chronic obstructive pulmonary disease; hsCRP, high-sensitivity C-reactive protein; IL-6, interleukin-6; LDL, low-density lipoprotein; HDL, high-density lipoprotein; LMR, lymphocyte-to-monocyte ratio.

**Table 3:** Stepwise multivariate logistic regression analysis including interleukin-6 (IL-6) for the composite endpoint (major amputation, MACE, or all-cause mortality).

Variable	OR (95% CI)	*p* Value
Age > 72 years	2.44 (1.22–4.89)	0.012
Malnutrition	4.25 (2.11–8.54)	<0.001
IL-6 above median	2.90 (1.45–5.81)	0.003

CI: confidence interval; IL-6: interleukin six; OR: odds ratio.

**Table 4:** Stepwise multivariate logistic regression analysis including high-sensitivity C-reactive protein (hsCRP) for the composite endpoint (major amputation, MACE, or all-cause mortality).

Variable	OR (95% CI)	*p* Value
Age > 72 years	2.19 (1.08–4.46)	0.03
Malnutrition	4.68 (2.26–9.68)	<0.001
hsCRP above median	4.22 (2.04–8.72)	<0.001

CI: confidence interval; hsCRP: high-sensitivity C-reactive protein; OR: odds ratio.

## Data Availability

De-identified data are available on request preceded by a signed data access agreement form.
